# Beyond the Senses: How Self-Directed Speech and Word Meaning Structure Impact Executive Functioning and Theory of Mind in Individuals With Hearing and Language Problems

**DOI:** 10.3389/fpsyg.2021.646181

**Published:** 2021-03-30

**Authors:** Thomas F. Camminga, Daan Hermans, Eliane Segers, Constance T. W. M. Vissers

**Affiliations:** ^1^Behavioural Science Institute, Radboud University, Nijmegen, Netherlands; ^2^Royal Dutch Kentalis, Sint-Michielsgestel, Netherlands

**Keywords:** self-directed speech, inner speech, word meaning structure, language, executive functioning, theory of mind, developmental language disorder, deaf and hard of hearing

## Abstract

Many individuals with developmental language disorder (DLD) and individuals who are deaf or hard of hearing (D/HH) have social–emotional problems, such as social difficulties, and show signs of aggression, depression, and anxiety. These problems can be partly associated with their executive functions (EFs) and theory of mind (ToM). The difficulties of both groups in EF and ToM may in turn be related to self-directed speech (i.e., overt or covert speech that is directed at the self). Self-directed speech is thought to allow for the construction of non-sensory representations (i.e., representations that do not coincide with direct observation). Such non-sensory representations allow individuals to overcome the limits set upon them by the senses. This ability is constrained by the development of word meaning structure (i.e., the way words are understood). We argue that the greater ability to construct non-sensory representations may result in more enhanced forms of EF and ToM. We conclude that difficulties in EF, ToM, and social–emotional functioning in those with hearing and language problems may be accounted for in terms of word meaning impairments. We propose that word meaning structure and self-directed speech should be considered in assigning EF and ToM treatments to individuals with DLD and those who are D/HH.

## Introduction

Individuals with developmental language disorder (DLD) and those who are deaf or hard of hearing (D/HH) often have social–emotional problems in addition to their learning problem (Dammeyer, 2010; Marschark et al., 2015; Hubert-Dibon et al., 2016; Conti-Ramsden et al., 2018). Both groups have lower self-rated self-esteem, higher risk of being bullied, and more problems with maintaining peer relations (e.g., Fellinger et al., 2015 and van den Bedem et al., 2018, 2019). In addition, they show increased signs of aggression, depression, and anxiety (e.g., Kvam et al., 2006; Theunissen et al., 2014, and Brownlie et al., 2016). In both groups, these social–emotional problems have been linked to difficulties in executive functioning (EF; e.g., Pauls and Archibald, 2016 and Botting et al., 2017) and theory of mind (ToM; e.g., Meristo et al., 2007 and Nilsson and de López, 2016). Recently, the claim has been made that these EF and ToM difficulties may be due to delays in the development of self-directed speech (Aziz, 2015; Vissers and Hermans, 2018; Mulvihill et al., 2019; Vissers et al., 2020). Here, we extend this hypothesis, by arguing that language problems are reflected by a limited understanding of word meanings, constraining the potential of self-directed speech in supporting EF and ToM.

## The Interplay Between Self-Directed Speech, EF, and ToM in Individuals with Hearing and Language Problems

DLD is attributed to individuals who are delayed in their language development in the absence of a known biomedical etiology (Bishop et al., 2017). DLD is a heterogeneous disorder that may be characterized by various underlying neuropsychological deficits (Tomas and Vissers, 2019). It has a prevalence of 7–14% in children younger than 5 years (Law et al., 2017). Many individuals who are D/HH are impaired in all aspects of language relative to their normal peers and even in some cases to children with DLD (Tomblin et al., 2015; de Hoog, 2017). In addition to their language problems, both groups have difficulties in EF and ToM (e.g., Hintermair, 2013; Vugs et al., 2014). For example, EF problems are three to five times more common in children that are D/HH compared to typically developing children (Hintermair, 2013). Interestingly, EF and ToM problems are generally restricted to D/HH children with hearing parents rather than those with D/HH parents (Schick et al., 2007; Hall et al., 2017), suggesting that their language problems result from a mismatch between their perceptual abilities and those of their family (Hall et al., 2019). Thus, sharing language (or communication more generally), be it spoken or signed, appears to be an important factor in the development of EF and ToM. This is corroborated by longitudinal relationships between language, on the one hand, and EF and ToM, on the other (Milligan et al., 2007; Slot and von Suchodoletz, 2018). Note that these relations are likely bidirectional, as EF and ToM have also been shown to support language development (e.g., Loosli et al., 2012; for children with DLD see Sikora et al., 2019). Here, we theoretically explore only the mechanisms underlying the first direction of causality.

The dominant view of EFs holds that they are “general-purpose control mechanisms that modulate the operation of various cognitive subprocesses and thereby regulate the dynamics of human cognition (Miyake et al., 2000, p. 50).” In social–emotional functioning, EFs help individuals, for example, to restrain impulsive or inappropriate actions, to shift their attention away from negative stimuli, and to modify their goals and plans in the light of the needs, goals, impulses, and emotions of others (Vissers and Hermans, 2018). Three EFs, with distinct neuroanatomical substrates, are generally considered to be the core EFs: working memory updating (updating), inhibition of prepotent responses (inhibition), and mental set shifting (shifting).

ToM is defined as the ability to understand the behavior of others in mental terms (Premack and Woodruff, 1978). It is preceded by a complex developmental path that includes several precursors, such as the capacity for imitation, joint attention, and emotion recognition and understanding. Four-year-olds may learn that individuals can have false beliefs, which is taken as a hallmark of ToM (Wellman et al., 2001). Starting from the age of 7 years, children may learn to distinguish what is said from what is meant (e.g., sarcasm). ToM supports prosocial behavior, by importing considerations of the thoughts and feelings of other people into the decision-making process. Two dimensions of ToM with distinctive neuroanatomical underpinnings can be discerned (Westby and Robinson, 2014). Cognitive ToM refers to reflections based on thoughts, beliefs, and intentions, whereas affective ToM concerns reflections on feelings and emotions (e.g., Dvash and Shamay-Tsoory, 2014). These reflections may be directed to one's own mental states (intrapersonal ToM) or those of others (interpersonal ToM; Tine and Lucariello, 2012).

A potential explanatory account for the difficulties of individuals with language problems in EF and ToM was suggested by Vygotsky and Luria (1930/1994). These authors traced the origins of self-directed speech to the social dialogue. They observed in their experiments that when children tried to get a desired object that was out of reach, they asked the experimenter for help. When the experimenter left the room, however, the children continued speaking about the object and their own behavior toward it, but now to themselves. In children around the age of 6 years, self-directed speech typically starts to internalize (it “goes underground;” Vygotsky, 1934/1986, p. 34) until it finally becomes silent (i.e., inner speech; Vygotsky, 1934/1986; Bivens and Berk, 1990; Damianova et al., 2012). In children with language problems, this internalization process appears to be delayed (i.e., inner speech and private speech emerge at a later age; Lidstone et al., 2012; Aziz et al., 2017), and they draw upon it to a lesser degree in planning (Kuvalja et al., 2014; Larson et al., 2019).

Self-directed speech—including its equivalent in sign language, self-directed signing—is universal among humans (e.g., Al Namlah et al., 2006; Zimmermann and Brugger, 2013; Thibodeaux et al., 2019), although its frequency and manner of application may vary between individuals and tasks (Alderson-Day and Fernyhough, 2015). The development of self-directed speech is thought to be completely intertwined with that of other cognitive functions such as EF and ToM (e.g., Newton and de Villiers, 2007; Lidstone et al., 2010, 2012). More precisely, self-directed speech can, under the influence of the social environment, be synthesized with (precursors of) EF and ToM into functional systems, which have properties that none of these cognitive functions have on their own (Vygotsky and Luria, 1930/1994; Fernyhough, 2010; Toomela, 2016). For example, false belief understanding has been hypothesized to emerge as a result of (social) activity-driven developments (e.g., incidental learning; Marschark and Knoors, 2014) in cognitive functions as diverse as elementary forms of ToM (e.g., joint attention; Tomasello, 2019), EF (e.g., shifting between perspectives of self and other), and language (as a representational format; Frye et al., 1995; Fernyhough, 2008).

## Beyond the Senses: Non-sensory Representations and Word Meaning Structure

According to Vygotsky and Luria (1930/1994) and Toomela (2016), the development of self-directed speech grounds new ways of relating to the mind and the external world. Words—or more generally, symbols (including signs)—are linked to referents, which are mental images that correspond (directly or indirectly) to an aspect of the world (de Saussure, 1966). Importantly, symbols can be brought in contexts that are not possible for their referents (Toomela, 2016). However, even though symbols can be used in the absence of their referents, symbol and referent still form a holistic unity in the mind—the activation of either the symbol or its referent results in the activation of the other (de Saussure, 1966). Consider, for example, the sentence “a giraffe is having a picnic at the bottom of the ocean.” This fictional sentence may evoke a mental image of a giraffe even if there is no giraffe in the direct environment. Thus, humans are able to temporarily ignore the immediate sensory present (Vygotsky and Luria, 1930/1994), and to construct non-sensory representations: mental descriptions or images of objects or events that do not coincide with, or even contradict, the immediately sensed present. Therefore, self-directed speech allows to represent the non-sensory world (i.e., aspects of the world that cannot be observed through the sensory organs; Toomela, 2016), as well as events and phenomena that are observable in principle, but not at the moment.

These properties of self-directed speech have important consequences for EF and ToM. However, the potential for constructing non-sensory representations is constrained by language development. In addition, based on earlier suggestions by Vygotsky (1934/1986) and Luria (1976), Toomela (2003, 2020b) proposed that word meaning structure (i.e., the way words are understood) may be especially relevant in this respect. Word meaning structure is explicitly related to qualitative developments in the potential for articulating certain types of verbal contents and fits with Vygotsky's functional systems approach. Thus, this article focuses on word meaning structure, without denying the roles of other language aspects (e.g., vocabulary, syntax, and pragmatics) in EF and ToM that have been pointed out by other researchers (e.g., Harris, 2005; Milligan et al., 2007; Müller et al., 2009)^1^.

**Figure 1:**
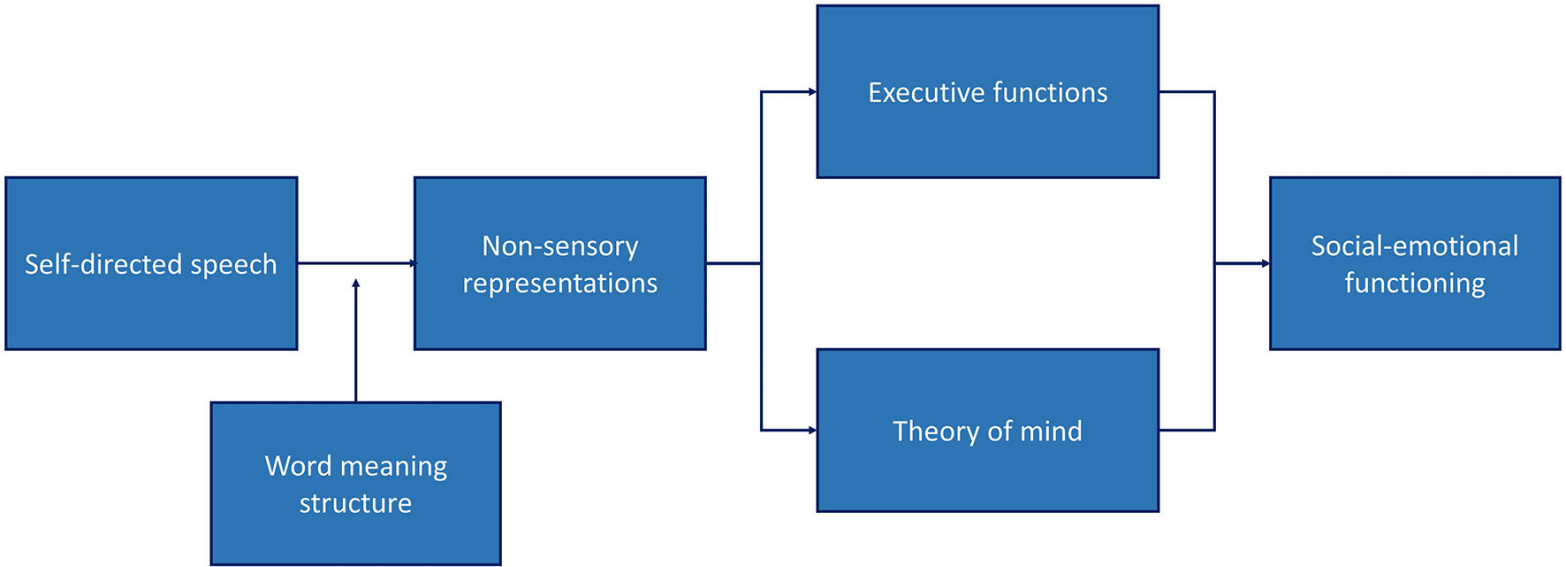
A Vygotskian account of the social–emotional problems of individuals with hearing and language problems. Social emotional functioning can be explained in terms of executive functioning and theory of mind, which in turn are affected by non-sensory representations created in self-directed speech. Non-sensory representations are images or descriptions corresponding to external events that do not coincide with direct observation. The ability to construct non-sensory representations is constrained by the level of word meaning structure.

Toomela (2003, 2020b) distinguishes five word meaning stages (Table 1). They are organized hierarchically, meaning that later stages emerge on the basis of earlier stages. Moreover, word meaning development is domain-specific, meaning that developments in one area do not guarantee improvements in other domains. In the first word meaning stage, that of *syncretic concepts*^2^, words have no fixed relation to their referent. A child may use a single word to refer to different aspects of a situation (an object, a property, or the whole situation), depending on the context. Next, in the stage of *object concepts*, two classes of words are differentiated, namely, words that refer to objects and object-specific properties. In the third stage, *everyday concepts*, children can implicitly learn all grammatical classes, allowing children to describe situations (i.e., relations between objects). In this stage, all aspects of the sensory world can be represented, as well as aspects of the non-sensory world, and even references to the past and the future. Categories that are signified by everyday concepts still have fuzzy boundaries, meaning that things can belong to categories in different degrees. Categorizations at this stage are based mainly on perceptual similarity and everyday activities. *Logical concepts*^3^, in contrast, are related to each other in a hierarchical taxonomy. Because of this hierarchical structure, logical concepts allow for a conscious differentiation of thought processes from the objects of thought and to group phenomena based on non-sensory properties. Moreover, logical concepts are characterized by categories with sharp, verbally defined boundaries that allow for abstract reasoning. Finally, *systemic concepts* embed these sharp, verbally defined categories within a broader system, as it can be realized that one object can belong to multiple categories, depending on the context (Figure 1).

**Table 1:** Overview of the stages of word meaning structure (WMS) and the development in executive functioning (EF) and theory of mind (ToM) that they allow for.

**WMS stages**					**WMS in EF**	**WMS in ToM**
	**Age**	**Referent**	**What can be described**	**Example: “whale”**		
Syncretic concepts	From 1 year old	Relation to referent is not fixed in any way, the referent can change depending on the context.	Aspects of the world can be labeled.	“Whale” may refer to a whale, one of its properties (e.g., a whale cry) or its context (the sea).	Labeling stimuli and representing them in working memory in the absence of their referent.	Verbally labeling emotions in the body, and facial expressions in others.
Object concepts	From 1.5 years old	Objects and object-specific properties. Objects are usually defined by their shape.	Properties can be verbally attributed to objects.	“Whale” refers to the shape of a whale.	Specific labeling and representation of (absent) objects and their properties.	Attributing emotions to specific agents.
Everyday concepts	From 3 years old	Objects, object-specific properties, and relations between objects (i.e., situations).	All aspects of the sensory world, as well as non-sensory aspects and fantasy worlds (understood in concrete, everyday terms).	“Whales are big and they swim in the sea.”	Representing verbal plans consisting of several consecutive steps that span into the far future.	Perspective taking; passing false belief tasks; attributing cognitive states, understood in a concrete, everyday manner.
Logical concepts	From 7 years old	Sharp linguistically defined categories based on necessary and sufficient attributes, or other (subordinate) words.	Coherent and logical understanding of the sensory and non-sensory world.	“A ‘whale’ is a mammal, because female whales have mammary glands.”	Creating precise and coherent verbal plans based on an accurate understanding of the non-sensory world	Coherent and potentially accurate understanding of mental states and underlying processes.
Systemic concepts	From 12 years old (if ever)	Sharp linguistically defined categories that are defined explicitly in relation to each other, but one object may belong to multiple categories depending on the context.	Valid understanding of sensory and non-sensory world whereby premises of conclusions are consciously selected and justified.	It is understood that a whale can be categorized as either a mammal or a fish, depending on the definition.	Greater ability to contextualize plans to specific circumstances in the context of larger goals	Understanding mental states in the context of the mind as a whole system.

*Word meaning development lays the basis for the corresponding EF and ToM development, but the latter may need more time to develop. The mammal example is based on Toomela (2003)*.

## A Vygotskian Perspective on EF Development: Links with the Stages of Word Meaning Structure

Vygotsky and Luria (1930/1994) showed that symbols can mediate the influences of the environment on individuals, thereby allowing individuals to regulate their cognitive processes and behavior. Here we argue that word meaning structure may play a unique role in EF, by constraining the potential for self-directed speech to construct non-sensory representations. In order to understand the problems of those with language and hearing problems in EF, we will propose advancements in the components of EF, namely, updating, inhibition, and shifting (Miyake et al., 2000), which result from each new word meaning stage.

### Syncretic and Object Concepts

Syncretic concepts and object concepts allow individuals to verbally label a stimulus. Verbal labels can be decoupled from their referents, resulting in an enduring trace that can be represented in working memory even in the referent's absence (Luria, 1962/1980; Al Namlah et al., 2006; Müller et al., 2009). Labeling thus allows individuals to bring established action programs in novel situations. Moreover, labels single out essential, and inhibit inessential aspects of the environment (Luria, 1961; Toomela, 2002; Müller et al., 2004). Finally, verbal labels have been shown to enhance shifting abilities (Jacques, 2001).

### Everyday Concepts

The opportunities for regulating behavior increase drastically with the emergence of everyday concepts. Luria (1962/1980) states that inner speech “plays an active part in […] singling out the aim of the action and providing a general scheme for it (p. 292).” In other words, self-directed speech can dictate an action plan that can be maintained in working memory in the face of changing environments. The inhibition of goal-irrelevant behaviors and information can be based on these verbal plans (Luria, 1962/1980). Verbal plans may mediate cases where behavioral patterns come into conflict. Interestingly, it is not until the age of 4 years that children can shift successfully on the flexible item selection task (FIST; Jacques, 2001) and the dimensional change card sorting (DCCS) task (Zelazo et al., 1996)—two tasks that involve shifting between conflicting verbal response rules.

### Logical and Systemic Concepts

By thinking in logical and systemic concepts, individuals can represent more coherent, accurate, and precise verbal plans that should facilitate more efficient inhibition and shifting. For example, a verbal rule such as “I will not look at my phone for the next hour” presupposes a sharp delineation of the word “not,” which is not supported by everyday conceptual thinking. Indeed, in a task that involves the memorization of two separate lists of words, Toomela et al. (2020) found that participants thinking predominantly in logical concepts were less susceptible to interference than those thinking in everyday concepts. Moreover, in a second task, the logical-conceptual thinkers had fewer difficulties in regulating their behavior in the face of potential interference.

## A Vygotskian Perspective on ToM Development: Links with the Stages of Word Meaning Structure

Many abilities linked to ToM, such as imitation and joint attention, can be coordinated without self-directed speech (Tomasello, 2019). However, thoughts and feelings cannot be observed directly through the senses (Premack and Woodruff, 1978). Therefore, explicit ToM—the ability to attribute mental states to oneself and others—requires, from the Vygotskian point of view (Vygotsky and Luria, 1930/1994; Toomela, 2016), the involvement of self-directed speech. Consequently, we argue that the development of the dimensions of ToM (cognitive, affective, interpersonal, intrapersonal) is constrained by the level of word meaning structure.

### Syncretic and Object Concepts

Syncretic concepts allow children to label their sensory experiences (object concepts add precision). Regarding affective ToM, these may include bodily sensations associated with one's own emotions, and facial expressions and body posture in others. Overtly or covertly saying labels brings the referents into awareness (Kolk, 2012; Toomela, 2016). However, these labels can initially only be connected to referents associated with observable phenomena (Toomela, 2020a). Therefore, the meanings of early mental state words may differ from those used by older children and adults (Booth et al., 1997). Still, labeling may facilitate aspects of ToM that emerge before the age of 3 years (Westby and Robinson, 2014), such emotion recognition, altruistic behavior, and prediction of the behavior of others, by making important aspects in the body and the environment more salient.

### Everyday Concepts

Fernyhough (2008) suggested that young children come to understand mental states, not through metacognitive inference, but by representing perceptual, epistemic, and affective perspectives of oneself and others in self-directed speech. The ability to verbally describe a situation from another person's perspective and to differentiate it from one's own can emerge on the basis of everyday concepts. For example, false belief understanding, a hallmark of cognitive ToM, emerges after the age of 4 years (Wellman et al., 2001). Linguistic devices such as complementation syntax (de Villiers and de Villiers, 2014) and contrastives (Wellman, 2014) may allow individuals to mentally differentiate between perspectives of oneself and others.

### Logical and Systemic Concepts

Logical concepts, through their hierarchical organization, support individuals in distinguishing their thought processes from the objects of thought, which indeed appears to be difficult for young children (e.g., Lagattuta et al., 2015 and Flavell et al., 1986). Thus, logical concepts may allow individuals to distinguish logical and probable from illogical and improbable inferences of mental states and processes. This may facilitate aspects of higher-order ToM, such as comprehension of lies, sarcasm, and figurative language, which emerge from 7 years old (Westby and Robinson, 2014). By increasing awareness of mental processes, logical concepts allow individuals to influence mental states (both cognitive and affective) more effectively in themselves and others (Vygotsky, 1934/1986). For example, awareness of negative cognitive distortions, as is facilitated by cognitive therapy, allows individuals to mitigate their influences on well-being (Leahy, 2017).

## EF and ToM Problems in Relation to Word Meaning Structure in Individuals with Hearing and Language Problems

As word meaning belongs to the language system (Vygotsky, 1934/1986; Luria, 1962/1980), it seems likely that individuals with hearing and language problems are delayed in their word meaning development. If so, this may explain their problems in their EF and ToM.

### Problems in EF

Deficits in EF are already present in preschoolers with DLD (Vissers et al., 2015) and preschoolers who are D/HH (Beer et al., 2014). Early EF deficits may be related to problems in regulating mental processes with verbal labels. Three-year-olds typically start subjecting their behavior to verbal plans. From this age, differences emerge between children with DLD and their typically developing peers on tasks that involve shifting between conflicting verbal response rules, such as the FIST (Roello et al., 2015) and the DCCS (Farrant et al., 2012). In older children and adolescents with hearing and language problems, EF deficits may be related to logical concept acquisition. Indeed, children with language problems have difficulties in operating with taxonomic information, a hallmark of logical conceptual thought (e.g., Marinellie and Johnson, 2002; Marschark et al., 2004, and Dosi and Gavriilidou, 2020).

### Problems in ToM

Deficits in cognitive and affective forms of social understanding are already evident in preschoolers with DLD (Vissers and Koolen, 2016) and preschoolers who are D/HH (e.g., Meristo et al., 2007 and Wiefferink et al., 2013). From the age of 3 years, clear differences emerge between children with hearing and language problems and their peers on false belief tasks (e.g., Meristo et al., 2007 and Nilsson and de López, 2016), and sociodramatic play (e.g., Cornelius and American, 1990 and Brown et al., 1997). This suggests that they find it harder to see the world from another person's perspective, indicating delayed everyday conceptual ToM development. No research exists yet on logical conceptual ToM in individuals with DLD and in those who are D/HH, but given the indications for problems with logical conceptual thinking, high performance in this domain seems unlikely.

## Conclusion

To conclude, we have aimed at achieving a better understanding of the social–emotional difficulties of individuals with DLD and those who are D/HH. We have argued that these individuals may have a less advanced word meaning structure, resulting in a limited potential for self-directed speech to support EF and ToM with non-sensory representations.

Strong conclusions are prohibited by a paucity of direct research on the topic. Future experimental and longitudinal research should assess whether this mechanism may indeed account for the social–emotional and academic problems of individuals with language problems (and *vice versa*). Thereby, it will be important to assess whether problems occur at the level of word meaning development or at the level of applying the learned linguistic concepts in concrete tasks (i.e., self-directed speech)^4^. One clinically relevant way to test this mechanism is by assessing whether word meaning structure relates to the effectiveness of treatments for individuals with DLD or individuals who are D/HH. For example, young children may benefit most from verbal labeling training (Jacques, 2001), whereas older children may benefit more from verbal planning for EF development (Abdul Aziz et al., 2016) and sociodramatic play (Qu et al., 2015) and metacognitive training for their ToM development. Through its mechanistic explanation of the interplay between language, EF, and ToM, the present framework could be used to improve existing EF and ToM treatments for individuals with hearing and language problems and to assign these treatments to specific stages of (word meaning) development.

## Author Contributions

Based on earlier work by CV and DH, TC has created the theoretical model described in the text. All authors have contributed to finalizing the perspective article.

## Conflict of Interest

The authors declare that the research was conducted in the absence of any commercial or financial relationships that could be construed as a potential conflict of interest.

## Footnotes

^1^The concepts of self-directed speech and word meaning structure should be clearly distinguished, because two individuals in the same word meaning stage may differ in the way they construct non-sensory representations in particular situations in self-directed speech.

^2^These stages concern verbal concepts (not preverbal, sensory-based concepts). However, for Vygotsky, the development of linguistic concepts is completely intertwined with cognitive development more broadly.

^3^Logical concepts correspond to Vygotsky's (1934/1986) “scientific concepts.”

^4^Especially in the latter case, other aspects of language, such as grammar, vocabulary, and pragmatics, should be considered as well.

## References

[B1] Abdul Aziz S. Fletcher J. Bayliss D. M. (2016). The effectiveness of self-regulatory speech training for planning and problem solving in children with specific language impairment. J. Abnorm. Soc. Psychol. 44, 1045–1059. 10.1007/s10802-015-0115-7 26678398

[B2] Al Namlah A. S. A.-N. Fernyhough C. Meins E. (2006). Sociocultural influences on the development of verbal mediation: private speech and phonological recoding in Saudi Arabian and British samples. Dev. Psychol. 1, 117–131. 10.1037/0012-1649.42.1.117 16420122

[B3] Alderson-Day B. Fernyhough C. (2015). Inner speech: development, cognitive functions, phenomenology, and neurobiology. Psychol. Bul. 141, 931–965. 10.1037/bul0000021 26011789 PMC4538954

[B4] Aziz S. A. (2015). Self-talk during planning and problem solving in young children with specific language impairment (Doctoral Thesis). University of Western Australia, Perth, WA, Australia.

[B5] Aziz S. A. Fletcher J. Bayliss D. M. (2017). Self-regulatory speech during planning and problem-solving in children with SLI and their typically developing peers. Int. J. Lang. Commun. Disord. 52:311–322. 10.1111/1460-6984.12273 27511872

[B6] Beer J. Kronenberger W. G. Castellanos I. Colson B. G. Henning S. C. Pisoni D. B. (2014). Executive functioning skills in preschool-age children with cochlear implants. ASHA 57, 1521–1534. 10.1044/2014_JSLHR-H-13-0054 24686747 PMC4190832

[B7] Bishop D. V. M. Snowling M. J. Thompson P. A. Greenhalgh T. The CATALISE-2 consortium (2017). Phase 2 of CATALISE: a multinational and multidisciplinary Delphi consensus study of problems with language development: terminology. J. Child Psychol. Psychiatry Allied Discip. 58, 1068–1080. 10.1111/jcpp.12721 28369935 PMC5638113

[B8] Bivens J. E. Berk L. E. (1990). A longitudinal study of the development of elementary school children's private speech. Merrill-Palmer Q. 66, 443–463.

[B9] Booth J. R. Hall W. S. Robison G. C. Kim S. Y. (1997). Acquisition of the mental state verb know by 2- to 5-year-old children. J. Psycholinguist. Res. 26, 581–603. 10.1023/A:1025093906884 9394459

[B10] Botting N. Jones A. Marshall C. Denmark T. Atkinson J. Morgan G. (2017). Nonverbal executive function is mediated by language: a study of deaf and hearing children. Child Dev. 88, 1689–1700. 10.1111/cdev.12659 27859007 PMC6849701

[B11] Brown P. M. Prescott S. J. Rickards F. W. Paterson M. M. (1997). Communicating about pretend play: a comparison of the utterances of 4-year-old normally hearing and deaf or hard-of-hearing children in an integrated kindergarten. Volta Rev. 1, 5–17.

[B12] Brownlie E. B. Bao L. Beitchman J. (2016). Childhood language disorder and social anxiety in early adulthood. J. Abnorm. Soc. Psychol. 44, 1061–1070. 10.1007/s10802-015-0097-5 26530522

[B13] Conti-Ramsden G. Durkin K. Toseeb U. Botting N. Pickles A. (2018). Education and employment outcomes of young adults with a history of developmental language disorder. Int. J. Lang. Commun. Disord. 53, 237–255. 10.1111/1460-6984.12338 29139196 PMC5873379

[B14] Cornelius G. American D. H. (1990). The play behavior of hearing-impaired kindergarten children. Ann. Deaf 135, 316–321. 10.1353/aad.2012.0542 2270824

[B15] Damianova M. K. Lucas M. Sullivan G. B. (2012). Verbal mediation of problem solving in pre-primary and junior primary school children. South Afr. J. Psychol. 42, 445–455. 10.1177/008124631204200316

[B16] Dammeyer J. (2010). Empirical articles psychosocial development in a Danish population of children with cochlear implants and deaf and hard-of-hearing children. J. Deaf Stud. Deaf Educ. 15, 50–58. 10.1093/deafed/enp024 19745003

[B17] de Hoog B. (2017). Spoken Language Development in children with cochlear implants. Radboud University.

[B18] de Saussure F. (1966). Course in General Linguistics, 3rd Edn. New York, NY: McGraw-Hill.

[B19] de Villiers J. G. de Villiers P. A. (2014). The role of language in theory of mind development. Top. Lang. Disord. 34, 313–328. 10.1097/TLD.000000000000003711405572

[B20] Dosi I. Gavriilidou Z. (2020). The role of cognitive abilities in the development of definitions by children with and without developmental language disorder. J. Psycholinguist. Res. 49, 761–777. 10.1007/s10936-020-09711-w 32592117

[B21] Dvash J. Shamay-Tsoory S. G. (2014). Theory of mind and empathy as multidimensional constructs. Top. Lang. Disord. 34, 282–295. 10.1097/TLD.0000000000000040

[B22] Farrant B. M. Maybery M. T. Fletcher J. (2012). Language, cognitive flexibility, and explicit false belief understanding: longitudinal analysis in typical development and specific language impairment. Child Dev. 83, 223–235. 10.1111/j.1467-8624.2011.01681.x 22188484

[B23] Fellinger M. J. Holzinger D. Aigner M. Beitel C. Fellinger J. (2015). Motor performance and correlates of mental health in children who are deaf or hard of hearing. Dev. Med. Child Neurol. 57, 942–947. 10.1111/dmcn.12814 26062643

[B24] Fernyhough C. (2008). Getting Vygotskian about theory of mind: mediation, dialogue, and the development of social understanding. Dev. Rev. 28, 225–262. 10.1016/j.dr.2007.03.001

[B25] Fernyhough C. (2010). Vygotsky, luria, and the social brain, in Self- and social regulation: Exploring the Relations Between Social Social Interaction, Cognition, and the Development of Executive Functions (Oxford: Oxford University Press), 56–79. 10.1093/acprof:oso/9780195327694.003.0003

[B26] Flavell J. H. Green F. L. Flavell E. R. (1986). Development of knowledge about the appearance-reality distinction. Monogr. Soc. Res. Child Dev. 51, 1–87. 10.2307/1165866 3807927

[B27] Frye D. Zelazo P. D. Palfai T. (1995). Theory of mind and rule-based reasoning. Cogn. Dev. 10, 483–527. 10.1016/0885-2014(95)90024-1

[B28] Hall M. L. Eigsti I. M. Bortfeld H. Lillo-Martin D. (2017). Auditory deprivation does not impair executive function, but language deprivation might: evidence from a parent-report measure in deaf native signing children. J. Deaf Stud. Deaf Educ. 22, 9–21. 10.1093/deafed/enw054 27624307 PMC5189172

[B29] Hall M. L. Hall W. C. Caselli N. K. (2019). Deaf children need language, not (just) speech. First Lang. 39, 367–395. 10.1177/014272371983410223526836

[B30] Harris P. L. (2005). Conversation, pretense, and theory of mind, in Why Language Matters for Theory of Mind (Oxford: Oxford University Press), 70–83. 10.1093/acprof:oso/9780195159912.003.0004

[B31] Hintermair M. (2013). Executive functions and behavioral problems in deaf and hard-of-hearing students at general and special schools. J. Deaf Stud. Deaf Educ. 18, 344–359. 10.1093/deafed/ent003 23418367

[B32] Hubert-Dibon G. Bru M. Gras Le Guen C. Launay E. Roy A. (2016). Health-related quality of life for children and adolescents with specific language impairment: a cohort study by a learning disabilities reference center. PLoS ONE 11:e0166541. 10.1371/journal.pone.0166541 27851795 PMC5112866

[B33] Jacques S. (2001). The roles of labelling and abstraction in the development of cognitive flexibility. University of Toronto.

[B34] Kolk H. (2012). Vrije wil is geen illusie: Hoe de hersenen ons vrijheid verschaffen. Bert Bakker.

[B35] Kuvalja M. Verma M. Whitebread D. (2014). Patterns of co-occurring non-verbal behaviour and self-directed speech; a comparison of three methodological approaches. Metacognition Learn. 9, 87–111. 10.1007/s11409-013-9106-7

[B36] Kvam M. H. Loeb M. Tambs K. (2006). Mental health in deaf adults: symptoms of anxiety and depression among hearing and deaf individuals. J. Deaf Stud. Deaf Educ. 12, 1–7. 10.1093/deafed/enl015 16950865

[B37] Lagattuta K. H. Kramer H. J. Kennedy K. Hjortsvang K. Goldfarb D. Tashjian S. (2015). Beyond Sally's missing marble. Adv. Child. Dev. Behav. 48, 185–217. 10.1016/bs.acdb.2014.11.005 25735945

[B38] Larson C. Gangopadhyay I. Kaushanskaya M. Weismer S. E. (2019). The relationship between language and planning in children with language impairment. J. Speech Lang. Hear. Res. 62, 2772–2784. 10.1044/2019_JSLHR-L-18-0367 31343936 PMC6802909

[B39] Law J. Charlton J. Dockrell J. Gascoigne M. McKean C. Theakston A. (2017). Early language development: Needs, provision, and intervention for preschool children from socioeconomically disadvantaged backgrounds.

[B40] Leahy R. (2017). Cognitive Therapy Techniques: A Practitioner's Guide, 2nd Edn. New York, NY: Guilford Press.

[B41] Lidstone J. S. M. Meins E. Fernyhough C. (2010). The roles of private speech and inner speech in planning during middle childhood: evidence from a dual task paradigm. J. Exp. Child Psychol. 107, 438–451. 10.1016/j.jecp.2010.06.002 20633894

[B42] Lidstone J. S. M. Meins E. Fernyhough C. (2012). Verbal mediation of cognition in children with specific language impairment. Dev. Psychopathol. 24, 651–660. 10.1017/S0954579412000223 22559137

[B43] Loosli S. V. Buschkuehl M. Perrig W. J. Jaeggi S. M. (2012). Working memory training improves reading processes in typically developing children. Child Neuropsychol. 18, 62–78. 10.1080/09297049.2011.575772 21623483

[B44] Luria A. R. (1961). The role of speech in the regulation of normal and abnormal behaviour. New York, NY: Liveright Publishing.

[B45] Luria A. R. (1962/1980). Higher Cortical Functions in Man. New York, NY: Basic Books. 10.1007/978-1-4615-8579-4

[B46] Luria A. R. (1976). Cognitive Development: Its Cultural and Social Foundations. Cambridge, MA: Harvard University Press.

[B47] Marinellie S. A. Johnson C. J. (2002). Definitional skill in school-age children with specific language impairment. J. Commun. Disord. 35, 241–259. 10.1016/S0021-9924(02)00056-4 12064786

[B48] Marschark M. Convertino C. McEvoy C. Masteller A. (2004). Organization and use of mental lexicon by deaf and hearing individuals. Am. Ann. Deaf 149, 51–61. 10.1353/aad.2004.0013 15332467

[B49] Marschark M. Knoors H. (2014). Teaching Deaf Learners Psychological and Developmental Foundations. Oxford: Oxford University Press.

[B50] Marschark M. Shaver D. M. Nagle K. M. Newman L. A. (2015). Predicting the academic achievement of deaf and hard-of-hearing students from individual, household, communication, and educational factors. Except. Child. 81, 350–369. 10.1177/0014402914563700 26549890 PMC4634639

[B51] Meristo M. Falkman K. W. Surian L. Siegal M. (2007). Language access and theory of mind reasoning: evidence from deaf children in bilingual and oralist environments. Dev. Psychol. 43, 1156–1169. 10.1037/0012-1649.43.5.1156 17723042

[B52] Milligan K. Astington J. W. Dack L. A. (2007). Language and theory of mind: meta-analysis of the relation between language ability and false-belief understanding. Child Dev. 78, 622–646. 10.1111/j.1467-8624.2007.01018.x 17381794

[B53] Miyake A. Friedman N. P. Emerson M. J. Witzki A. H. Howerter A. Wager T. D. (2000). The unity and diversity of executive functions and their contributions to complex “frontal lobe” tasks: a latent variable analysis. Cogn. Psychol. 41, 49–100. 10.1006/cogp.1999.0734 10945922

[B54] Müller U. Jacques S. Brocki K. Zelazo P. D. (2009). The executive functions of language in preschool children, in Private Speech, Executive Functioning, and the Development of Verbal Self-Regulation, eds Winsler A. Fernyhough C. Montero I., (Cambridge: Cambridge University Press), 53–68. 10.1017/CBO9780511581533.005

[B55] Müller U. Zelazo P. D. Hood S. Leone T. Rohrer L. (2004). Interference control in a new rule use task: age-related changes, labeling, and attention. Child Dev. 5, 1594–1609. 10.1111/j.1467-8624.2004.00759.x 15369533

[B56] Mulvihill A. Carroll A. Dux P. E. Matthews N. (2019). Self-directed speech and self-regulation in childhood neurodevelopmental disorders: current findings and future directions. Dev. Psychopathol. 32, 205–217. 10.1017/S0954579418001670 30704545

[B57] Newton A. M. de Villiers J. G. (2007). Thinking while talking: adults fail nonverbal false-belief reasoning. Psychol. Sci. 18, 574–579. 10.1111/j.1467-9280.2007.01942.x 17614864

[B58] Nilsson K. K. de López K. J. (2016). Theory of mind in children with specific language impairment: a systematic review and meta-analysis. Child Dev. 87, 143–153. 10.1111/cdev.12462 26582261

[B59] Pauls L. J. Archibald L. M. D. (2016). Executive functions in children with specific language impairment: a meta-analysis. J. Speech Lang. Hear. Res. 59, 1074–1086. 10.1044/2016_JSLHR-L-15-0174 27653611

[B60] Premack D. Woodruff G. (1978). Does the chimpanzee have a theory of mind? Behav. Brain Sci. 1, 515–526. 10.1017/S0140525X0007651218424224

[B61] Qu L. Shen P. Chee Y. Y. Chen L. (2015). Teachers' theory-of-mind coaching and children's executive function predict the training effect of sociodramatic play on children's theory of mind. Soc. Dev. 24, 716–733. 10.1111/sode.12116

[B62] Roello M. Ferretti M. L. Colonnello V. Levi G. (2015). When words lead to solutions: executive function deficits in preschool children with specific language impairment. Res. Dev. Disabil. 37, 216–222. 10.1016/j.ridd.2014.11.017 25528081

[B63] Schick B. de Villiers P. A. de Villiers J. G. Hoffmeister R. (2007). Language and theory of mind: a study of deaf children. Child Dev. 78, 376–396. 10.1111/j.1467-8624.2007.01004.x 17381779

[B64] Sikora K. Roelofs A. Hermans D. Knoors H. (2019). Executive control in language production by children with and without language impairment. Int. J. Lang. Commun. Disord. 54, 645–655. 10.1111/1460-6984.12470 30920093

[B65] Slot P. L. von Suchodoletz A. (2018). Bidirectionality in preschool children's executive functions and language skills: is one developing skill the better predictor of the other? Early Child. Res. Q. 42, 205–214. 10.1016/j.ecresq.2017.10.005

[B66] Theunissen S. C. P. M. Rieffe C. Kouwenberg M. De Raeve L. J. I. Soede W. Briaire J. J.. (2014). Behavioral problems in school-aged hearing-impaired children: the influence of sociodemographic, linguistic, and medical factors. Eur. Child Adolesc. Psychiatry 23, 187–196. 10.1007/s00787-013-0444-4 23807768

[B67] Thibodeaux J. Bock A. Hutchison L. Winsler A. (2019). Singing to the self: children's private speech, private song, and executive functioning. Cogn. Dev. 50, 1–260. 10.1016/j.cogdev.2019.04.005

[B68] Tine M. Lucariello J. (2012). Unique theory of mind differentiation in children with autism and asperger syndrome. Autism Res. Treat. 2012, 1–11. 10.1155/2012/505393 22934174 PMC3420603

[B69] Tomas E. Vissers C. (2019). Behind the scenes of developmental language disorder: time to call neuropsychology back on stage. Front. Hum. Neurosci. 12:517. 10.3389/fnhum.2018.00517 30687040 PMC6333853

[B70] Tomasello M. (2019). Becoming Human: A Theory of Ontogeny. Cambridge, MA: Belknap Press. 10.4159/9780674988651

[B71] Tomblin J. B. Harrison M. Ambrose S. E. Walker E. A. Oleson J. J. Moeller M. P. (2015). Language outcomes in young children with mild to severe hearing loss. Ear Hear. 36, 76S−91S. 10.1097/AUD.0000000000000219 26731161 PMC4704115

[B72] Toomela A. (2002). Drawing as a verbally mediated activity: a study of relationships between verbal, motor, and visuospatial skills and drawing in children. Int. J. Behav. Dev. 26, 234–247. 10.1080/01650250143000021

[B73] Toomela A. (2003). Development of symbol meaning and the emergence of the semiotically mediated mind, in Cultural Guidance in the Development of the Human Mind, ed Toomela A. (New York, NY: Aplex), 163–209.

[B74] Toomela A. (2016). What are higher psychological functions? Integr. Psychol. Behav. Sci. 50, 91–121. 10.1007/s12124-015-9328-0 26403987

[B75] Toomela A. (2020a). Culture, speech, and my self . Väike Vanker.

[B76] Toomela A. (2020b). Studies in the mentality of literates: searching for the cultural great divide at the individual level of analysis. Integr. Psychol. Behav. Sci. 54, 1–29. 10.1007/s12124-019-09503-5 31641930

[B77] Toomela A. Filho D. B. Bastos A. C. S. Chaves A. M. Ristum M. Chaves S. S.. (2020). Studies in the mentality of literates: 2. Conceptual structure, cognitive inhibition and verbal regulation of behavior. Integr. Psychol. Behav. Sci. 54, 880–902. 10.1007/s12124-020-09517-4 32125602

[B78] van den Bedem N. P. Dockrell J. E. Van Alphen P. M. Kalicharan S. V. Rieffe C. (2018). Victimization, bullying, and emotional competence: longitudinal associations in (pre)adolescents with and without developmental language disorder. J. Speech Lang. Hear. Res. 61, 2028–2044. 10.1044/2018_JSLHR-L-17-0429 29998317

[B79] van den Bedem N. P. Willems D. Dockrell J. E. van Alphen P. M. Rieffe C. (2019). Interrelation between empathy and friendship development during (pre)adolescence and the moderating effect of developmental language disorder: a longitudinal study. Soc. Dev. 28, 599–619. 10.1111/sode.12353

[B80] Vissers C. Hermans D. (2018). Social-emotional problems in deaf and hard-of-hearing children from an executive and theory-of-mind perspective, in Evidence-Based Practices in Deaf Education, eds Knoors H. Marschark M. (Oxford: Oxford University Press), 437–454. 10.1093/oso/9780190880545.003.0020

[B81] Vissers C. Koolen S. (2016). Theory of mind deficits and social emotional functioning in preschoolers with specific language impairment. Front. Psychol. 7:1734. 10.3389/fpsyg.2016.01734 27867370 PMC5095688

[B82] Vissers C. Koolen S. Hermans D. Scheper A. Knoors H. (2015). Executive functioning in preschoolers with specific language impairment. Front. Psychol. 6:1574. 10.3389/fpsyg.2015.01574 26539136 PMC4611093

[B83] Vissers C. Tomas E. Law J. (2020). The emergence of inner speech and its measurement in atypically developing children. Front. Psychol. 11:279. 10.3389/fpsyg.2020.00279 32256423 PMC7090223

[B84] Vugs B. Hendriks M. Cuperus J. Verhoeven L. (2014). Working memory performance and executive function behaviors in young children with SLI. Res. Dev. Disabil. 35, 62–74. 10.1016/j.ridd.2013.10.022 24240018

[B85] Vygotsky L. S. (1934/1986). Thought and Language. Cambridge, MA: MIT Press.

[B86] Vygotsky L. S. Luria A. R. (1930/1994). Tool and symbol in child development, in The Vygotsky Reader, eds van der Veer R. Valsiner J. (Oxford: Blackwell Publishing), 99–174.

[B87] Wellman H. M. (2014). Making Minds: How Theory of Mind Develops. Oxford: Oxford University Press. 10.1093/acprof:oso/9780199334919.001.0001

[B88] Wellman H. M. Cross D. Watson J. (2001). Meta-analysis of theory-of-mind development: the truth about false belief. Child Dev. 72, 655–684. 10.1111/1467-8624.00304 11405571

[B89] Westby C. Robinson L. (2014). A developmental perspective for promoting theory of mind. Top. Lang. Disord. 34, 362–382. 10.1097/TLD.0000000000000035

[B90] Wiefferink C. H. Rieffe C. Ketelaar L. De Raeve L. J. I. Frijns J. H. M. (2013). Emotion understanding in deaf children with a cochlear implant. J. Deaf Stud. Deaf Educ. 18, 175–186. 10.1093/deafed/ens042 23232770

[B91] Zelazo P. D. Frye D. Rapus T. (1996). An age-related dissociation between knowing rules and using them. Cogn. Dev. 11, 37–63. 10.1016/S0885-2014(96)90027-1

[B92] Zimmermann K. Brugger P. (2013). Signed soliloquy: visible private speech. J. Deaf Stud. Deaf Educ. 18, 261–270. 10.1093/deafed/ens072 23325673

